# Modern Concepts in Cardiovascular Disease: Inflamm-Aging

**DOI:** 10.3389/fcell.2022.882211

**Published:** 2022-05-18

**Authors:** Yustina M. Puspitasari, Stefano Ministrini, Lena Schwarz, Caroline Karch, Luca Liberale, Giovanni G. Camici

**Affiliations:** ^1^ Center for Molecular Cardiology, University of Zurich, Schlieren, Switzerland; ^2^ Internal Medicine, Angiology and Atherosclerosis, Department of Medicine and Surgery, University of Perugia, Perugia, Italy; ^3^ First Clinic of Internal Medicine, Department of Internal Medicine, University of Genoa, Genoa, Italy; ^4^ IRCCS Ospedale Policlinico San Martino Genoa—Italian Cardiovascular Network, Genoa, Italy; ^5^ Department of Cardiology, University Heart Center, University Hospital Zurich, Zurich, Switzerland; ^6^ Department of Research and Education, University Hospital Zurich, Zurich, Switzerland

**Keywords:** inflammaging, cardiovascular disease, aging, inflammation, senescence

## Abstract

The improvements in healthcare services and quality of life result in a longer life expectancy and a higher number of aged individuals, who are inevitably affected by age-associated cardiovascular (CV) diseases. This challenging demographic shift calls for a greater effort to unravel the molecular mechanisms underlying age-related CV diseases to identify new therapeutic targets to cope with the ongoing aging "pandemic". Essential for protection against external pathogens and intrinsic degenerative processes, the inflammatory response becomes dysregulated with aging, leading to a persistent state of low-grade inflammation known as inflamm-aging. Of interest, inflammation has been recently recognized as a key factor in the pathogenesis of CV diseases, suggesting inflamm-aging as a possible driver of age-related CV afflictions and a plausible therapeutic target in this context. This review discusses the molecular pathways underlying inflamm-aging and their involvement in CV disease. Moreover, the potential of several anti-inflammatory approaches in this context is also reviewed.

## Introduction

Life expectancy is steadily increasing due to improvements in medical treatment and living conditions, resulting in a growing portion of elderly individuals. Aging is an inevitable biological phenomenon characterized by a progressive deterioration of physiological functions. Consequently, aging is globally recognized as an independent risk factor for different diseases, including those of the CV system. Thus, the ever-increasing number of elderly people will eventually result in a “pandemic” of age-dependent CV disorders (Camici and Liberale, 2017). A better understanding of the specific role of aging in the development of CV pathologies and the molecular mechanisms underlying this association is, therefore, a matter of top priority.

Under physiological conditions, inflammation protects against external pathogens and intrinsic degenerative processes (Chen et al., 2018). Nevertheless, dysregulation of the immune system, as seen during aging, triggers a persistent state of low-grade inflammation, which has been recognized as an important driver for the development of age-related diseases (Ferrucci and Fabbri, 2018; Liberale et al., 2022; Tansey et al., 2022). This phenomenon, referred to as inflamm-aging, has been linked to a higher risk of CV events and has been increasingly recognized as a determinant of CV outcome (Liberale et al., 2020), This review summarizes the current knowledge about inflamm-aging and its involvement in the pathogenesis of age-related CV diseases. Finally, the potential of inflammation as a therapeutic target is also discussed based on available experimental and clinical evidence.

## Molecular Determinants of Inflamm-Aging

**FIGURE 1:**
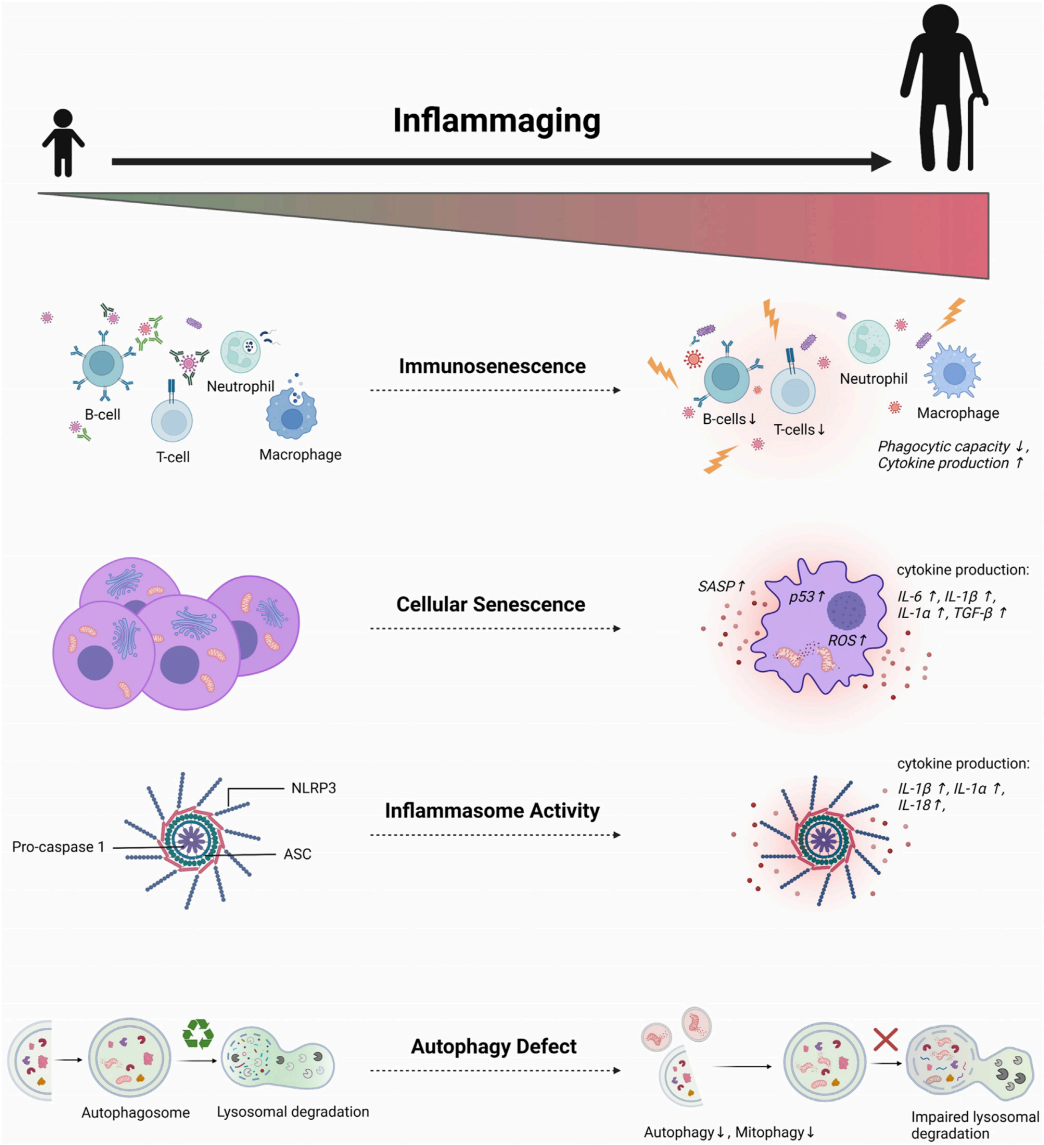
Multifactorial mechanisms of inflamm-aging. Several molecular pathways are involved in triggering age-related chronic low-grade inflammation. Aging affects both innate and adaptive immune systems, leading to sustained low-grade immune activation and reduced sensitivity to appropriate immunogenic stimuli. Such deterioration contributes to inadequate senescent cell clearance. Consequently, the accumulation of senescent cells induces chronic exposure to inflammatory SASP proteins. Aberrant inflammasome activation also occurs in aging due to defects in autophagy and mitophagy, thus further perpetuating the age-related pro-inflammatory milieu. SASP = senescence-associated secretory phenotype; ROS = Reactive oxygen species; NLRP3 = Nod-like receptor protein 3; ASC = Apoptosis speck-like protein; IL = Interleukin (Created with BioRender.com)

Inflamm-aging was firstly theorized by Franceschi et al. in the 2000s as a phenomenon involved in the age-related deterioration of physiological processes. Defined as a chronic low-grade sterile inflammation, inflamm-aging was suggested to result from persistent antigenic load and stress (Franceschi et al., 2000). Since then, this concept has been intensively studied to identify its molecular mechanisms and how it contributes to age-dependent diseases. Even though the exact mechanisms of inflamm-aging are not yet fully elucidated, some pathologic features have been identified (Figure 1).

### Cellular Senescence

Senescence is a phase of cell cycle, characterized by an irreversible replicative arrest (Olivieri et al., 2018; Sanada et al., 2018). Firstly reported by Hayflick and Moorhead, this phenomenon is a hallmark of aging and is driven by various intrinsic and extrinsic stressors, such as telomere shortening (Hayflick and Moorhead, 1961). As non-coding repetitive nucleotide sequences are located at the end of chromosomes, telomeres prevent chromosomal end-to-end fusion and instability (Vaiserman and Krasnienkov, 2021). In proliferating cells, telomeres shorten gradually at each cell division until they reach a critical length. Short telomeres cause chromosomal fusion and activation of transcription factor p53, ultimately leading to replicative senescence (RS) and apoptosis. (Toussaint et al., 2002; de Magalhães and Passos, 2018). On the other side, stress-induced premature senescence (SIPS) is a cellular phenotype developing in response to endogenous and exogenous stressors, such as oxidative stress, inflammation, and irradiation (Raghuram and Mishra, 2014), independently of telomere shortening (De Magalhães et al., 2002).

Being involved in physiological processes such as tissue regeneration and tumor suppression (Prata et al., 2018), senescence is increasingly considered a fundamental contributor to inflamm-aging. Indeed, senescent cells display a senescence-associated secretory phenotype (SASP), characterized by increased expression and release of pro-inflammatory cytokines, chemokines, and proteases, such as Interleukin (IL)-6, IL-1β, IL-1α, and TGF-β through NFkB (Van Deursen, 2014; Ferrucci and Fabbri, 2018; Rea et al., 2018; Docherty et al., 2020).

Molecular features of SASP are extremely heterogenous, depending on cell types and inducing factors. Nevertheless, the physiological goal of SASP is activating the immune cells to promote the clearance of senescent cells (Docherty et al., 2020). Aging of the immune system results in an inadequate elimination of senescent cells, which exponentially accumulate in multiple organs and thereby lead to a sustained pro-inflammatory condition (Ferrucci and Fabbri, 2018; Rea et al., 2018). Accordingly, accelerated senescence and an increased number of senescent cells characterize different chronic degenerative diseases, including atherosclerosis, cancer, and heart failure (Stojanovic et al., 2020).

### Immunosenescence

Immunosenescence refers to a wide range of age-associated disorders of the immune system. Affecting both innate and adaptive immune responses, immunosenescence impact all functions of the immune system including defense against pathogens and aberrant cells, and long-term immunity (Weiskopf et al., 2009; Shaw et al., 2013). As a consequence, the elderly are exposed to a higher risk of acute infections, re-activation of chronic infections, cancers and vaccine failure (Weiskopf et al., 2009).

Innate immunity represents the front-line host defense. It consists of different components, including physical barriers, effector cells, and soluble mediators, providing a fast primary response against invading pathogens (Hato and Dagher, 2015). Aging affects all the components of innate immunity (Weiskopf et al., 2009). In particular, reduced phagocytic capacity is considered the hallmark of immunosenescence. An impairment of the respiratory burst, a pivotal mechanism in intracellular pathogen destruction, has been observed in neutrophils of both aged mice and elderly individuals (Davila et al., 1990; Butcher et al., 2001; Weiskopf et al., 2009; Shaw et al., 2013; Takahashi et al., 2016; Ventura et al., 2017; Lloberas et al., 2019). In addition, natural killer (NK) cells, which are crucial in suppressing viral infection and tumor cells, display an age-related dysfunction, despite the increased number (Weiskopf et al., 2009; Gounder et al., 2018). In detail, aging is associated with impaired response of NK cells to IL-2, reduced IFN- γ secretion and degranulation, resulting in the attenuation of their cytotoxic function (Beli et al., 2011; Camous et al., 2012; Shaw et al., 2013). Finally, dendritic cells (DCs) display impairment of antigen uptake and presentation to T cells. Monocyte-derived DCs from aged individuals are less effective than those of younger subjects in inducing T cell proliferation and production of IFN-γ (Agrawal et al., 2017). Indeed, the ability of DCs to prime CD8^+^ T cells in the elderly was found to be inadequate (Briceño et al., 2016). Nevertheless, the underlying mechanisms of the impact of age on DC function are still not fully understood and to this end, additional studies will be required.

Aging also affects the adaptive immune system. T cells maturate within the thymus, an organ undergoing a volume reduction and a functional decay alongside aging (Weiskopf et al., 2009), especially thymopoiesis and the central tolerance process. As a result, T cells maturation is impaired, leading to depletion of naïve T cells and increase of self-reactive T cells, thus facilitating autoimmune responses (Thomas et al., 2020). Similar to T cells, the number and function of B cells are both reduced in the elderly. B cells produce antibodies with a lower affinity toward antigens (Weiskopf et al., 2009), and this could be due to the deficient expression of CD28 co-stimulatory molecule on T cells (Boucher et al., 1998) (Weiskopf et al., 2009; Paudel et al., 2019; Pangrazzi and Weinberger, 2020). CD28^−^ T cells have a pro-inflammatory phenotype, and the accumulation of CD8^+^CD28^−^ T cells in elderly individuals correlates with a higher risk of age-related disease and mortality (Pangrazzi and Weinberger, 2020).

Hematopoietic stem cells (HSCs) -a key component of this process-maintain the production of all the lineage of blood cells in the body, including myeloid and lymphoid cells (Aw et al., 2007; Sawai et al., 2016). However, aging leads to their exhaustion resulting in lower capacity for self-renewal and deterioration of their differentiation ability, with significant consequences on both adaptive and innate immunity. Furthermore, with age, HSCs accumulate mutagenic events, which lead to positive selection and outgrowth of selected clones (Khetarpal et al., 2019; Mooney et al., 2021). The presence of clonal mutant stem cells without the development of overt hematologic malignancy and other clonal diseases is referred to as clonal hematopoiesis of indeterminate potential (CHIP) (Tyrrell and Goldstein, 2021; Libby and Ebert, 2018; Khetarpal et al., 2019). The most frequently encountered mutated genes in CHIP are DNA methyltransferase 3 (DNMT3A), ten-eleven-translocation 1 (TET2), Janus kinase 2 (Jak2), and additional sex comb-like 1 (ASXL1) (Jaiswal et al., 2014; Mooney et al., 2021). A cross-sectional study reported that the frequency of clonal somatic mutations in peripheral blood cells increases with age, reaching a prevalence of around 10% in individuals above 70 years, whereas they are rarely found in younger individuals (Jaiswal et al., 2014). Immune effector cells derived from mutated stem cells are functionally altered and favor a pro-inflammatory milieu (Leoni et al., 2017). Indeed, individuals with clonal hematopoiesis show higher circulating IL-6, TNF-α, and MCP-1 compared to age-matched individuals (Mooney et al., 2021).

### NLRP3 Inflammasome Activity

The Nod-like receptor protein 3 (NLRP3) inflammasome complex consists of NLRP3, apoptosis speck-like protein (ASC), and pro-caspase 1. Upon stimulation, NLRP3 inflammasome activates caspase-1, which cleaves the precursors of inflammatory cytokines, such as IL-1β, IL-1α, and IL-18 (Martinon et al., 2009; Rea et al., 2018; Liu et al., 2020). The activation of NLRP3 inflammasome consists of two essential steps named priming and triggering. NLRP3 priming is ignited by stress signals, including endogenous danger-associated molecular patterns (DAMPs) and exogenous pathogen-associated molecular patterns (PAMPs) molecules, which results in NF-κB activation and the consequent upregulation of NLRP3, which is otherwise low in unstimulated cells (Boaru et al., 2015; Afonina et al., 2017; Kelley et al., 2019). Upregulation of NLRP3 promotes a cascade of events (triggering), leading to the assembly of the NLRP3 inflammasome complex and its activation (Afonina et al., 2017; Kelley et al., 2019). In the absence of NLRP3 and ASC, old healthy animals present lower expression of IL-1β and IL-18 than the control group, suggesting the involvement of NLRP3 inflammasome in inflamm-aging (Youm et al., 2013). This age-related NLRP3 activation could be due to the accumulation of DAMPs along with aging, including adenosine triphosphate, uric acid, and cholesterol crystals (Youm et al., 2013).

Autophagy and mitophagy are intracellular processes dedicated to the removal of dysfunctional cytoplasmic materials and misfolded proteins and, ultimately, regulate inflammasome activation. (Barbosa et al., 2019; Biasizzo and Kopitar-Jerala, 2020). Autophagy activity is compromised in aging due to impaired epigenetic and transcriptional regulation of autophagy genes; thus, progressive accumulation of damaged organelles typically occurs (Wong et al., 2020). Mitophagy–the selective removal process of damaged mitochondria–has a major role in age-related diseases, including those of the CV system. Mitophagy preserves mitochondrial integrity and quality control along with mitochondrial fission and fusion (Chen et al., 2020). The accumulation of Parkin, an essential protein that amplifies the polyubiquitination of mitochondrial surface proteins to initiate mitophagy, is detected in aged cardiomyocytes, suggesting defective mitophagy in the aging heart (Thai et al., 2019; Ajoolabady et al., 2020). Mitophagy is impaired in aging as a result of downregulation of crucial elements, such as PTEN-induced kinase (PINK)-1 (Ajoolabady et al., 2020).

Previous studies have identified the involvement of mtROS and mtDNA, which are released by damaged mitochondria, in triggering NLRP3 inflammasome activation (Zhou et al., 2011; Elliott and Sutterwala, 2015; Grazioli and Pugin, 2018; Biasizzo and Kopitar-Jerala, 2020). Interestingly, this mechanism leads to a vicious circle, where caspase-1 further inhibits mitophagy, thus amplifying the mitochondrial damage. (Yu et al., 2014). Oxidized mtDNA was demonstrated to directly bind to NLRP3, resulting in NLRP3 inflammasome activation and increased IL-1β production (Shimada et al., 2012). Furthermore, mtROS induce NLRP3 inflammasome activity also indirectly *via* NF-κB pathway (Ferrucci and Fabbri, 2018).

## Inflamm-Aging and CV Diseases

Chronic low-grade inflammation levels, characterizing inflamm-aging, have been increasingly recognized as a crucial aspect of different CV diseases. Indeed, evidence indicates the contribution of the inflammatory process to the pathogenesis and outcome of age-related CV diseases (Ruparelia et al., 2017; Ferrucci and Fabbri, 2018; Liberale et al., 2018, 2020; Bonetti et al., 2019). As research in this field progresses, the impact of inflamm-aging and its underlying mechanism on CV diseases begins to be elucidated.

### Atherosclerosis

As the underlying pathological process of many CV diseases, atherosclerosis shows a strong association with age, and it is extremely frequent in elderly individuals. The role of inflammation in this process and its interplay with modified lipids have been increasingly recognized (Libby, 2013; Stojanovic et al., 2020; Ministrini et al., 2021a).

Atherogenesis starts at a very young age with the accumulation of modified lipoproteins within the vessel wall and escalates with advanced age. The progression of atherosclerosis is facilitated by endothelial cell dysfunction due to increased ROS generation (Ross, 1999; Ruparelia et al., 2017; Tyrrell and Goldstein, 2021). Indeed, the dysfunctional endothelium favors the uptake of lipoproteins and the recruitment of immune cells in the vascular wall, accelerating plaque formation. (Galkina and Ley, 2007). Notably, a higher number of macrophages infiltrating the wall is detected in the atherosclerotic aortas of aged mice with hyperlipidemia compared to young animals (Du et al., 2016). Also, in comparison to young animals, aged mice on high-fat diet display higher levels of IL-6 and macrophage-attracting chemokines in their arteries (Du et al., 2016). Once beneath the endothelial layer, monocytes differentiate into macrophages, internalize modified lipoproteins and transform into foam cells. Accumulated apoptotic foam cells integrate into the lipid necrotic core of the atherosclerotic plaque and increase its instability (Ruparelia et al., 2017; Rea et al., 2018). Such a process further sustains a state of chronic unresolved inflammation, as the lesion itself contains antigens that contribute to increasing pro-inflammatory mediators. Among others, cholesterol crystals induce the activation of the NLRP3 inflammasome (Stojanovic et al., 2020). Moreover, senescent endothelial cells contribute to the higher NLRP3 activity due to upregulated oxidative stress and defect of autophagy (Rea et al., 2018).

As atherosclerosis advances, an increased number of senescent cells accumulates within the lesion. These cells express SASP, consisting of various pro-inflammatory cytokines, growth factors, and proteases, which exacerbate the inflammatory reaction, promote plaque growth and cause its destabilization (Childs et al., 2015; Stojanovic et al., 2020). For instance, metalloproteinases secreted by senescent cells digest the plaque’s fibrous cap, which becomes more prone to rupture (Libby, 2013). The pro-inflammatory milieu also results in an increased risk of thrombotic occlusion, the ultimate cause of most ischemic complications of atherosclerosis (Ferrucci and Fabbri, 2018; Soysal et al., 2020). Therefore, acting as one of the sources of chronic low-grade inflammation, senescent cells can be potential therapeutic targets to regulate atherosclerosis burden in the elderly (Stojanovic et al., 2020).

On the other hand, CHIP has also been proposed as a risk factor for atherosclerosis. In particular, mutations of TET2 are associated with inflammation and atherosclerosis. TET2-knockout atherosclerotic-prone animals develop larger aortic plaques than the control group (Fuster et al., 2017). Moreover, TET2 deficient macrophages respond to atherogenic stimuli such as LDL by releasing higher amounts of inflammatory mediators, such as IL-6, IL-1β, and IL-18 (Fuster et al., 2017; Jaiswal et al., 2017; Jaiswal and Ebert, 2019). Being involved in the DNA demethylation process, TET2 facilitates the suppression of IL-6 and IL-1β, inflammatory mediators implicated in the pathogenesis of atherosclerosis (Libby and Ebert, 2018; Cong et al., 2021). Indeed, the deleterious effect of TET2 knockdown can be reversed by treatment with the NLRP3 inhibitor MCC950, indicating the pivotal role of IL-1β/NLRP3 inflammasome in TET2 atherosclerosis-related signaling (Fuster et al., 2017).

### Myocardial Infarction

A regenerative process of injured tissue is crucial after myocardial infarction (MI) (Karin and Clevers, 2016; Appel et al., 2021). However, the accumulation of senescent cells in the heart reduces its resilience to cardiac stress, and a gradual deterioration in tissue repair is strongly associated with aging (Owens et al., 2021). Following an ischemic event, elderly individuals display an impaired vascular response, including angiogenesis, which is driven by the inflammatory response (Fan et al., 2013). Indeed, aged animals display an inadequate inflammatory response and cardiac repair after MI, with consequent adverse remodeling and increased mortality (Bujak et al., 2008). By eliminating dead cells and initiating the healing process, an aptly and effective inflammatory response is critical for favorable cardiac repair after MI and to prevent its long-term complications, such as heart failure (HF) (Frantz et al., 2013; Rieckmann et al., 2019) I dysfunctional activation of immune cells, characterizing inflamm-aging, has been associated with a poorer prognosis after MI in the elderly (Ma et al., 2018).

Accumulating evidence demonstrates a pivotal role for macrophages in the myocardial homeostasis, in both physiologic conditions and response to injury, as they orchestrate the repair response (Nahrendorf and Swirski, 2013; Swirski and Nahrendorf, 2013). As discussed above, macrophage phagocytic capacity is impaired in aging, resulting in an unbalanced inflammatory response post-MI impacting cardiac healing (Ma et al., 2018). Specifically, impaired phagocytosis is associated with the reduced clearance of dead cardiomyocytes (Bujak et al., 2008). Moreover, a deviation of cardiac-resident macrophage subpopulations in the aging heart has been reported, showing an increased M1 subset and a reduction of M2 macrophages (Ma et al., 2018). Although a wide functional overlap exists, M1 macrophages are generally considered the pro-inflammatory subset since they secrete pro-inflammatory cytokines (i.e., TNF-α, IL-6, and IL-1β) and MMPs. In contrast, M2 macrophages release anti-inflammatory cytokines (e.g., IL-10) and pro-reparative factors, such as TGF-β and vascular endothelial growth factor (VEGF), responsible for extracellular matrix deposition, healing, and fibrosis (Ma et al., 2018; O’Rourke et al., 2019). An increased proportion of M1-to-M2 macrophages, as observed in aging animals, results in infarct size augmentation and extensive extracellular matrix remodeling (O’Rourke et al., 2019).

Inflammasome activity is also pivotal for myocardial response to ischemia. ASC is prominently expressed in the myocardium of patients with ischemic heart disease (Kawaguchi et al., 2011). Indeed, NLRP3, IL-1β, and IL-18 gene expression are markedly upregulated in the infarcted heart (Sandanger et al., 2013). In line with this finding, *in vivo* deletion of caspase-1 and NLRP3 yields a cardioprotective effect with smaller infarct size and preserved cardiac function (Kawaguchi et al., 2011). The release of inflammatory cytokines (i.e., IL-1β and IL-18) following inflammasome activation further impacts cardiac resident cells, promoting apoptosis and fibrosis (Mauro et al., 2019).

### Heart Failure

Heart failure (HF) is the final stage of multiple structural and functional cardiac disorders, and it is one of the most common CV diseases among the elderly (Hoogen et al., 2019; Liu et al., 2020). Chronic inflammation and auto-immunity play an important role in the onset and progression of HF (Dick and Epelman, 2016; Hoogen et al., 2019), as demonstrated by the increased number of inflammatory cells, observed in the myocardium of chronic HF patients (Hoogen et al., 2019).

The role of aging as a risk factor for HF has been extensively investigated. Previous observation reported a dampened autophagy in the heart of aged animals (Xu et al., 2016). Moreover, increased telomere shortening and expression of senescence markers, such as p53 and p16^Ink4a^, were also observed in aging hearts, indicating a higher accumulation of senescent cells that might contribute to age-related cardiac dysfunction (Torella et al., 2004; Li et al., 2020). By developing a senescent phenotype, cardiomyocytes from old animals promote inflammation, cell death, and senescence in neighboring cells *via* SASP signaling (Li et al., 2020). Besides, nontypical SASP signals secretion from senescent cardiomyocytes has been observed to promote cardiac fibroblast activation. Indeed, clearance of senescent cells significantly reduces cardiac hypertrophy and fibrosis (Anderson et al., 2019).

Recently, the involvement of IL-1β in HF progression has been proposed. A previous *in vivo* study on outbred CD-1 mice demonstrated the induction of myocardial dysfunction by administration of recombinant IL-1β. Interestingly, the impairment was reversible, as the contractile dysfunction returned to baseline five days after the end of the treatment, suggesting the therapeutic potential for targeting IL-1β in HF (Van Tassell et al., 2013). Besides IL-1β, TNF-α, and IL-6 are involved in the pathogenesis of HF. Indeed, circulating levels of these cytokines predict survival in patients with HF (Moro-García et al., 2014). The increase of these pro-inflammatory cytokines was correlated to the increased expression of Toll-like receptor-4 (TLR-4)—similarly to what is observed in aging–both in the myocardium and in circulating monocytes from patients with chronic HF (Birks et al., 2004). Experimental models demonstrate a gradual increase in cardiac IL-6 expression along with aging. At the same time, its deficiency mitigates age-related cardiac dysfunction (Wang et al., 2020b). Interestingly, higher circulating levels of IL-6 have been reported in elderly patients with both HF and CHIP, particularly those with double somatic mutations of DNMT3A and TET2 genes (Pascual-Figal et al., 2021). Based on this observation, the involvement of age-related clonal hematopoiesis in the development of HF has been suggested. This notion is further supported by previous results from *in vivo* study showing that TET2 deficiency accelerates age-related cardiomyopathy with more pronounced cardiac hypertrophy and fibrosis (Wang et al., 2020a). Nonetheless, future studies will be required to investigate the role of CHIP in HF progression and its molecular mechanisms in more detail.

### Aortic Aneurysm

Aortic aneurysm (AA) refers to a pathologic dilation of the aortic wall up to 1.5 times its normal diameter, with a tendency to further expand and rupture (Hellawell et al., 2021). The mortality rate among patients with AA reaches 81%, including pre- and in-hospital deaths, thus underscoring its severe nature. Age is one of the most relevant risk factors for AA. Indeed, the risk of developing AA increases significantly, up to 40% above 65 years of age (Hellawell et al., 2021).

Described initially as a noninflammatory lesion, characterized by a loss of vascular smooth muscle cells and the fragmentation of vascular connective tissue, AA is nowadays considered the result of chronic inflammation in the vessel wall, which also involves inflammasome activity. Histological sections of AA show accumulation and infiltration of macrophages and lymphocytes in the aortic media and adventitia (Yuan et al., 2021), associated with a higher expression of ASC, caspase-1, and NLRP3 (Wortmann et al., 2021). Moreover, age-associated upregulation of *CASP1* and *IL1B* gene expression was also detected in peripheral blood mononuclear cells (PBMC) of patients with AA, suggesting a close relationship between AA and inflamm-aging (Wu et al., 2015). NLRP3 inflammasome activation in AA was proposed to be mediated by circulating homocysteine–a methionine-derived sulfur-containing amino acid whose levels increase with age (Kuo et al., 2005). Indeed, previous *in vivo* studies demonstrated an aggravation of AA following homocysteine supplementation by activation of adventitial fibroblast and NLRP3 inflammasome in macrophages (Liu et al., 2012; Sun et al., 2015).

## Future Perspective: Targeting Age-Related Inflammation in CV Disease

With mounting evidence about the crucial contribution of inflamm-aging in the pathogenesis of CV diseases, new therapeutic approaches targeting inflammatory molecular pathways have been increasingly investigated. Several clinical trials investigated the efficacy of anti-inflammatory compounds on CV diseases (Table 1). Unfortunately, elderly individuals are often under-represented in RCTs, resulting in an important lack of knowledge and missing the opportunity to implement personalized medicine.

**TABLE 1:** Clinical trials of anti-inflammatory agents in cardiovascular disease

Trial	Study population	Drug	Target	Dosage	Results
ARISE Tardif et al. (2008)	6’144 patients with ACS 14-365 days before randomization	Succinobucol	oxLDL	300 mg daily	Neutral: No reduction of CV events consisting of CV death, resuscitated cardiac arrest, non-fatal MI, non-fatal stroke, hospitalization for UA, and coronary revascularization
LoDoCo Nidorf et al. (2013)	532 patients with stable coronary disease	Colchicine	Microtubule assembly	0.5 mg daily	Beneficial: Significant reduction of CV events consisting of ACS, out-of-hospital cardiac arrest, and non-cardioembolic ischemic stroke
SELECT-ACS Tardif et al. (2013)	544 patients with NSTEMI scheduled for coronary angiography and possible ad hoc PCI.	Inclacumab	P-selectin	20 mg/kg, pre-procedural	Beneficial: Reduction in troponin I level and creatine kinase-MB from baseline at 16 and 24 h after PCI
VISTA-16 Nicholls et al. (2014)	5’145 patients with recent ACS	Varespladib	sPLA_2_	500 mg daily	Neutral: No reduction of risk of recurrent CV events consisting of CV death, non-fatal MI, non-fatal stroke, and UA with evidence of ischemia requiring hospitalization. Furthermore, increased risk of MI
STABILITY The STABILITY Investigators, (2014)	15’828 patients with stable coronary heart disease	Darapladib	Lp-PLA2	160 mg daily	Neutral: No reduction in risk for CV events consisting of CV death, MI, and stroke
SOLID-TIMI-52 O’Donoghue et al. (2014)	13’026 patients within 30 days of hospitalization with ACS	Darapladib	Lp-PLA_2_	160 mg daily	Neutral: No reduction in risk of major CV events consisting of coronary heart disease death, MI, and urgent coronary revascularization for myocardial ischemia
MRC-ILA Heart Study Morton et al. (2015)	182 patients with NSTE-ACS presenting <48 h from onset of chest pain	Anakinra	IL-1R antagonist	100 mg daily	Neutral: No reduction in MACE at 30 days and 3 months of treatment despite the significant reduction of inflammatory markers (CRP and IL-6) after 14 days treatment
SELECT-CABG Stähli et al. (2016)	384 patients randomized between 4 h and 6 weeks before CABG surgery to receive inclacumab	Inclacumab	P-selectin	20 mg/kg, 4-weeks intervals	Neutral: No reduction in saphenous vein graft disease after CABG
LATITUDE-TIMI-60 O’Donoghue et al. (2016)	3’503 patients hospitalized with acute MI and with at least 1 additional predictor for CV risk	Losmapimod	p38 MAP Kinase	7.5 mg twice daily	Neutral: No reduction of risk of CV events consisting of CV death, MI, and severe recurrent ischemia requiring urgent coronary revascularization
CANTOS Ridker et al. (2017)	10’061 patients with previous MI and hs-CRP ≥ 2 mg/L	Canakinumab	IL-1β	150 mg every 3 months	Beneficial: Significant reduction of recurrent CV events consisting of non-fatal MI, non-fatal stroke, and CV death
CIRT Ridker et al. (2019)	4’786 patients with previous MI or multivessel coronary disease who additionally had either T2DM or metabolic syndrome.	Methotrexate	Purinergic signalling	15-20 mg weekly	Neutral: No reduction of inflammatory mediators (IL-1β, IL-6, and CRP) and CV events consisting of non-fatal MI, non-fatal stroke, and CV death
COLCOT Tardif et al. (2019)	4’745 patients with acute MI in the past 30 days	Colchicine	Microtubule assembly	0.5 mg daily	Beneficial: Significant reduction of CV events consisting of CV death, resuscitated cardiac arrest, MI, stroke, and urgent hospitalization for angina leading to coronary revascularization
VCU-ART3 Abbate et al. (2020)	99 patients with STEMI	Anakinra	IL-1R	100 mg once or twice daily	Beneficial: Reduced incidence of HF and HF hospitalization with significant reduction of systemic inflammatory response
LoDoCo2 Nidorf et al. (2020)	5’522 patients with chronic coronary disease	Colchicine	Microtubule assembly	0.5 mg daily	Beneficial: Reduced risk of CV events consisting of composite of CV death, spontaneous MI, ischemic stroke, and ischemia-driven coronary revascularization
COPS Tong et al. (2020), Tong et al. (2021)	795 patients with ACS or evidence of CAD	Colchicine	Microtubule assembly	0.5 mg twice daily (1st month), once daily (for 11th month)	Neutral on the first year outcomes: No difference in primary outcome, higher rate in total death, in particular non-CV death. Beneficial on the second year outcome after cessation at 12 months: significant reduction in the primary endpoint consisting all-cause mortality, ACS, ischemia-driven urgent revascularization, and noncardioembolic ischemic stroke.

Given its important role in the progression of atherogenesis, MI and HF, IL-1 has emerged as a promising therapeutic target, leading to the investigation of different compounds targeting its signaling pathways, such as recombinant human IL-1 receptor antagonist (Anakinra) and human monoclonal anti-IL-1β antibodies (Canakinumab and Gevokizumab), in the setting of CV diseases (Buckley and Abbate, 2018; Liberale et al., 2021). The randomized double-blind trial Canakinumab Anti-inflammatory Thrombosis Outcomes Study (CANTOS) assessed the efficacy of canakinumab in preventing recurrent CV events in individuals with previous MI and persistent systemic inflammation (hsCRP level ≥2 mg/L). Canakinumab, administered subcutaneously every three months in addition to standard therapy, reduced hsCRP level by 41% in the highest dose arm (300 mg) compared to placebo. Further, canakinumab successfully reduced the incidence of primary endpoints (i.e., non-fatal MI, non-fatal stroke, or CV death) when administered at 150 or 300 mg at a median follow-up of 3.7 years (Ridker et al., 2017). However, canakinumab-treated patients exhibited neutropenia and showed an increased risk of fatal infections (Ridker et al., 2017), thus reducing the possibility to use such an approach for long-term treatments.

Meanwhile, promising outcomes were also observed with anakinra. With a shorter half-life (6 h), anakinra shows a better safety profile, as its dose can be easily managed to prevent adverse effects (Buckley and Abbate, 2018). Previous clinical studies in rheumatoid arthritis patients, with or without coronary artery disease, showed that a single dose of anakinra improves left ventricular and coronary vascular function (Ikonomidis et al., 2008; Ikonomidis et al., 2014). Blunted inflammatory response, defined as a reduction of hsCRP level, was also observed in ST-segment elevation MI and acute decompensated heart failure patients after 14 days of anakinra treatment (Abbate et al., 2013; Van Tassell et al., 2016). Nevertheless, secondary analysis of the MRC-ILA Heart Study showed that 14 days-long treatment with anakinra in non-ST-segment elevation MI patients does not improve long-term clinical outcomes. Instead, one year after anakinra discontinuation, a significant higher MACE incidence was observed in the treatment group, likely driven by a non-significant increase in recurrent myocardial infarction (Morton et al., 2015).

Even more promising results in secondary prevention of CV diseases were yielded by colchicine. The anti-inflammatory characteristics of colchicine are due to its inhibitory effect on microtubule formation. Through its effect, colchicine specifically affects different aspects of the inflammatory response, sparing from immune cell mobility to degranulation and including cell replication or cytokine release (Leung et al., 2015). Furthermore, colchicine also inhibits NLRP3 inflammasome polymerization (Martinon et al., 2009; Martínez et al., 2018). In 2013, the first Low Dose Colchicine (LoDoCo) trial was conducted on stable CAD patients, showing the beneficial effect of low-dose colchicine (0.5 mg/day) in reducing primary outcomes (i.e., the composite incidence of acute coronary syndrome, out-of-hospital cardiac arrest, or non-cardioembolic ischemic stroke) after 3-year median follow-up (Nidorf et al., 2013). However, LoDoCo was a relatively small trial. Thus, this study prompted additional trials. In 2019, the results of the large-scale Colchine Cardiovascular Outcomes Trial (COLCOT) confirmed its beneficial cardiovascular effect. At a median follow-up of 22.6 months, COLCOT demonstrated that low-dose colchicine reduces the risk of CV events in patients with a recent MI, mainly driven by a reduced incidence of stroke and urgent hospitalization for non-ST elevated MI or unstable angina (Tardif et al., 2019). More recently, the second LoDoCo trial (LoDoCo2) confirmed the encouraging results in the setting of chronic coronary disease, demonstrating a significantly lower occurrence of CV events in colchicine-treated patients compared to placebo (Nidorf et al., 2020). On the contrary, the Colchicine in Patients with Acute Coronary Study (COPS) multicenter trial reported no significant difference in primary endpoints between patients treated with low-dose oral colchicine and controls following acute coronary syndrome (ACS), whereas higher sepsis-related mortality in colchicine-treated patients was observed (Tong et al., 2020). Interestingly, a significant reduction in the primary end point in the colchicine group compared to the control was reported on the second year follow-up of this study after cessation at 12 months (Tong et al., 2021), suggesting the possible beneficial effect of the addition of low-dose colchicine to the standard medical therapy of ACS in the acute setting.

Regardless of the favorable outcomes of canakinumab and colchicine in reducing secondary CV events, other anti-inflammatory drugs show no beneficial effect. The Cardiovascular Inflammation Reduction Trial (CIRT) investigated methotrexate in patients with stable atherosclerosis against placebo and yielded neutral results, showing no effect on MACE incidence and IL-1β, IL-6, and hs-CRP levels (Ridker et al., 2019). Overall, the abovementioned results highlight the importance of understanding the specific contribution of the different inflammatory pathways in determining CV risk. Thus far, targeting the IL-1β pathway - either *via* direct inhibition or by intersecting the upstream signaling pathway - seems to be the most effective approach. Hence, NLRP3 inflammasome and caspase-1, the upstream mediators of IL-1β and several other inflammatory cytokines, are increasingly considered alternative therapeutical targets for CV prevention, and several inhibitory molecules against these proteins have been identified and are under investigation.

Recent *in vivo* studies have demonstrated the beneficial effect of MCC950 (a specific NLRP3 inhibitor) and VX-765 (a caspase-1 inhibitor) in a rodent model of MI, showing reduced infarct size, myocardial fibrosis and preserved cardiac function in MCC950 treated animals (Audia et al., 2018; Gao et al., 2019). Moreover, a reduced formation of atherosclerotic lesions presumably due to a reduction of macrophage influx was also observed in ApoE knockout mice treated with MCC950 for four weeks (Van Der Heijden et al., 2017). Collectively, these positive results highlight the potential of NLRP-3 and caspase-1 inhibitors as novel therapeutic agents for CV diseases and may set the stage for further investigations, particularly in advanced atherosclerosis.

Since senescent cells are the major source of inflammatory mediators in aging organisms, senolytics–a class of drugs that eliminates senescent cells–have been recently proposed as a pharmacological approach to prevent age-related CV afflictions, especially atherosclerosis. The first generation of senolytic drugs was identified from a transcriptome analysis in 2015 by Zhu et al., which included Dasatinib (D), a tyrosine kinase inhibitor, and Quercetin (Q), a flavonoid found in fruits and vegetables (Zhu et al., 2015; Ministrini et al., 2021b). Being approved for human use, both D and Q and their combination have been investigated as potential therapeutical compounds to treat age-related diseases (Kirkland and Tchkonia, 2020). A 3-months treatment with Dasatinib and Quercetin improves vascular function and calcification and increases p-eNOS^ser1177^ levels in aged mice (Roos et al., 2016). Navitoclax is another potential senolytic drug that inhibits the apoptosis regulator protein B-cell lymphoma 2 (Bcl-2) (Walaszczyk et al., 2019). Aged mice receiving an intermittent treatment with Navitoclax displayed preserved cardiac function and improved survival following MI. However, the potential of senolytic agents as a new therapeutic strategy is still hampered by adverse effects. For instance, evidence of cardiotoxicity was reported in previous clinical trials following Dasatinib administration as a treatment of chronic myeloid leukemia (Chaar and Kamta, 2018). Moreover, Navitoclax administration induces severe thrombocytopenia due to Bcl-XL inhibition_,_ which is essential for platelet survival (Schoenwaelder et al., 2011; Dookun et al., 2020).

Due to the substantial side effects displayed by different senolytics, an alternative approach targeting senescent cells using CAR T cells was recently proposed. Initially invented for cancer therapy, this approach utilizes genetically engineered T cells that express chimeric receptor proteins targeting senescence-specific surface antigens (Feucht and Abou-El-Enein, 2020). Senolytic CAR T cells have been tested in mice by targeting urokinase-type plasminogen activator receptor (uPAR), leading to the elimination of senescent cells and restoration of tissue homeostasis, thus demonstrating the therapeutic potential of this approach against age-associated disorders, including CV diseases (Amor et al., 2020; Feucht and Abou-El-Enein, 2020). Nevertheless, additional trials and investigations will be needed to translate senolytics application to the clinic, especially with respect to the identification of the best treatment strategy with minimum adverse effects (Kirkland and Tchkonia, 2020).

## Conclusion

Inflammation is a complex biological process with fundamental roles in host defense, tissue healing, and regeneration. However, the chronic low-grade activation of inflammatory pathways that characterize older individuals, the so-called inflamm-aging, has been associated with reduced lifespan and age-related CV disorders. Since CV disorders are the leading cause of death in industrialized countries, improving the treatment of these diseases implies prolonging the average lifespan. Furthermore, acute CV events, particularly stroke, are associated with long-term disability, inevitably resulting in a worsening of quality of life. Long-term disability, dependence on daily living, and reduced quality of life are the most relevant backlashes of aging; thus, addressing those aspects is pivotal in promoting healthy aging. Finally, inflamm-aging is also involved in other age-related disorders, like sarcopenia (Vatic et al., 2020), cancer (Park et al., 2021), and neurocognitive impairment(Komleva et al., 2021), all having a heavy impact on lifespan and quality of life of elderly people. Therefore, targeting inflamm-aging may prolong lifespan and promote successful aging by acting on multiple levels.

Inflamm-aging develops due to senescent cell accumulation, altered function of immune cells, and increased inflammasome activity due to incremented levels of DAMPs and PAMPs. To date, wide experimental evidence validated the importance of inflamm-aging in the pathophysiology of CV diseases and the potential of targeting inflammation as pharmacological therapy. Nevertheless, none of the tested anti-inflammatory agents has yet been implemented in everyday clinical cardiology; thus, more work remains to be done to optimize these promising interventions. Ultimately, future studies are encouraged to discover further potential therapeutic targets involved in the complex mechanism of inflamm-aging. Along with other treatment strategies against different age-related alterations in molecular pathways, inflamm-aging targeted approaches will intently endure the burden of CV disease in the growing aging population.
